# Nadir hemoglobin is associated with poor outcome from intracerebral hemorrhage

**DOI:** 10.1186/2193-1801-2-379

**Published:** 2013-08-13

**Authors:** Tiffany R Chang, Amelia K Boehme, Aimee Aysenne, Karen C Albright, Christopher Burns, T Mark Beasley, Sheryl Martin-Schild

**Affiliations:** Stroke Program, Department of Neurology, Tulane University School of Medicine, 1440 Canal St., TB-52, Suite 1000, 70112 New Orleans, USA; Divison of Neurosciences Critical Care, Department of Anesthesiology and Critical Care Medicine, School of Medicine, Johns Hopkins University, 21287 Baltimore, USA; Department of Epidemiology, School of Public Health, University of Alabama at Birmingham, 35294 Birmingham, England; Department of Neurology, School of Medicine, University of Alabama at Birmingham, 35294 Birmingham, England; Health Services and Outcomes Research Center for Outcome, Effectiveness Research and Education (COERE), Birmingham, England; Center of Excellence in Comparative Effectiveness Research for Eliminating Disparities (CERED), Minority Health & Health Disparities Research Center (MHRC), Birmingham, England; Department of Biostatistics, School of Public Health, University of Alabama at Birmingham, 35294 Birmingham, England

**Keywords:** Intracerebral hemorrhage, Anemia, Hemoglobin, Transfusion, Outcome

## Abstract

**Objective:**

Examine the relationship between anemia and outcomes from intracerebral hemorrhage (ICH).

**Methods:**

Patients admitted with spontaneous ICH between July 2008 and December 2010 were identified from our prospective stroke registry. Patients were divided into two groups based on admission hemoglobin (low vs. normal based on laboratory reference range for gender). Baseline characteristics were compared between groups using Chi-square, t-tests and Wilcoxon Rank Sum tests. Primary outcome was functional status at discharge, with modified Rankin Scale (mRS) 5–6 considered a poor outcome. Cumulative logit and logistic regression models were used to assess the relationships between baseline hemoglobin, nadir hemoglobin, and transfusion with outcomes.

**Results:**

Of the 109 patients, 28% (n = 30) were anemic on admission. Baseline anemia did not predict the primary outcome. Nadir hemoglobin was associated with poor functional outcome at discharge (OR = 1.58, 95% CI 1.31-1.90, p < 0.0001) and remained significant after adjusting for age, baseline NIHSS, transfusion, and length of stay (OR = 1.43, 95% CI 1.06-1.94, p = 0.02). Patients who received a transfusion had 9 times greater odds of having a discharge mRS 5–6 (OR 9.37, 95% CI 2.84-30.88, p = 0.0002) compared with patients who did not receive transfusion. This was no longer statistically significant after adjusting for other factors impacting outcome (OR 4.01, 95% CI 0.64-25.32, p = 0.1392). Neither nadir hemoglobin nor transfusion was found to be independent predictors of in-hospital mortality.

**Conclusion:**

This study suggests that nadir hemoglobin, not admission hemoglobin, can be used to predict poor functional outcome. Transfusion was not an independent predictor of poor outcome from ICH.

## Background

Anemia is a common problem in all critical care settings. The World Health Organization (WHO) defines anemia as hemoglobin less than 13 g/dl in men and 12 g/dl in women. (WHO/UNICEF/UNU 2001) A hemoglobin level between 7–9 g/dl is generally well-tolerated. Higher goals may be indicated in certain populations, such as patients with acute coronary syndromes. (Hebert et al. 1999) Although anemia can be corrected by red blood cell transfusion, liberal transfusion strategies in the intensive care unit have been associated with worse outcomes in a number of studies. (Hebert et al. 1999; Vincent et al. 2002; Corwin et al. 2004; Villanueva et al. 2013) Treatment goals for anemia in the neurocritical care unit remain controversial and vary depending on the nature of the neurological injury.

The presence of anemia is common in patients with intracerebral hemorrhage (ICH), occurring in as high as 72% of patients during hospitalization. (Sheth et al. 2011) Anemia has been associated with worse outcomes following ICH, subarachnoid hemorrhage (SAH), and traumatic brain injury (TBI). (Diedler et al. 2010; Naidech et al. 2006; Naidech et al. 2007; Kramer et al. 2009; Duane et al. 2008; Bussiere et al. 2013) A retrospective study of supratentorial ICH showed that patients with poor neurological outcomes (modified Rankin Scale, mRS 4–6) at discharge and at three months had lower average and nadir hemoglobin values during their hospital stay. (Diedler et al. 2010) Furthermore, anemia has been associated with increased hematoma size, (Kumar et al. 2009) which is a known predictor of ICH mortality. (Hemphill et al. 2001).

The effect of packed red blood cell (pRBC) transfusion to treat anemia on outcomes is not known. Specifically, there are conflicting reports regarding the association between transfusion and outcome after ICH, (Sheth et al. 2011; Diedler et al. 2010) and to our knowledge there is little published data evaluating the effect of aggressive treatment of anemia on hematoma volume or expansion following ICH.

The aim of this study was to examine the relationship between hemoglobin values, transfusion, and short-term outcomes in acute ICH patients. We hypothesized that admission hemoglobin, nadir hemoglobin, and pRBC transfusion would all be significant independent predictors of poor outcome in this patient population.

## Methods

We retrospectively reviewed consecutive patients entered into a prospectively collected ICH database who presented to our center between July 2008 and December 2010. Data was collected by trained abstractors via chart review. Patients were included in the study if they were 18 years of age or older, admitted to the Stroke Service, and had a final diagnosis of spontaneous ICH. Patients were excluded if ICH was found to be secondary to a vascular malformation, aneurysm, or underlying mass lesion. Following the initial assessment, patients underwent computed tomography (CT) scan and other imaging studies as needed. The exposures of interest were admission hemoglobin, nadir hemoglobin, and pRBC transfusion. The decision to administer pRBC transfusion was made based on physician judgment and was not standardized. Patients were divided into two groups, normal and low baseline hemoglobin, based on gender specific WHO reference ranges. (WHO/UNICEF/UNU 2001) The primary outcome of interest was functional status at discharge, based on modified Rankin Scale (mRS). All mRS scores were determined prospectively by a mRS-certified attending vascular neurologist. Secondary outcome measures included discharge National Institute of Health Stroke Scale (NIHSS), length of stay, and in-hospital mortality. All NIHSS scores were determined by NIHSS-certified examiners.

Baseline characteristics were evaluated using Chi-square, t-test and Wilcoxon Rank Sum tests. Functional outcome at discharge was classified as good (mRS 0–2), moderate (mRS 3–4) or poor (mRS 5–6). Cumulative logit and logistic regression models were used to assess the relationships between baseline hemoglobin, nadir hemoglobin, and transfusion and outcomes. Models were adjusted for variables that were either clinically or statistically significant at baseline. This study was approved by the Tulane University Institutional Review Board.

## Results

### Admission hemoglobin

A total of 109 patients with acute ICH were included in the analysis. Baseline patient characteristics are shown in Table 1. Low hemoglobin was present in 30 patients (28%) at admission. Admission hemoglobin was the nadir hemoglobin in 10 patients (9.2%). There were no significant differences in age, gender, race, smoking, antithrombotic use, NIHSS, GCS, ICH score, or ICH location between the patients with low and normal hemoglobin. Patients with low hemoglobin had lower median diastolic blood pressure than non-anemic patients (98.5 mmHg vs. 105 mmHg; p = 0.014). Nadir hemoglobin was significantly lower in patients with decreased admission hemoglobin compared with patients with normal hemoglobin (8.7 g/dl vs. 12 g/dl; p < 0.0001). The two groups had similar rates of transfusion with fresh frozen plasma and platelets to minimize hematoma expansion. There was no difference in hematoma volume expansion at 24 hours after admission between the two groups.

**Table 1:** **Baseline patient characteristics**

	Low hemoglobin n = 30	Normal hemoglobin n = 79	p value
Age, median (range)	56.5 (27–89)	61 (30–90)	0.103
Male, %	60	51.9	0.448
Race			0.544
White, %	26.7	27.8	
Black, %	66.7	68.4	
Asian, %	3.3	2.5	
Hispanic, %	0	1.3	
Active Smoker, %	23.3	33.3	0.312
Antithrombotic at home, %	40	34.6	0.602
History of Stroke/TIA, %	16.7	25.3	0.337
History of Hypertension, %	76.7	82.3	0.507
Baseline SBP mmHg, median (range)	170 (115–280)	195 (70–252)	0.186
Baseline DBP mmHg, median (range)	98.5 (56–140)	105 (45–170)	**0.014**
Baseline NIHSS, median (range)	16 (0–40)	14 (1–40)	0.287
GCS, median (range)	12.5 (3–15)	14 (3-15	0.504
Admit ICH Score	1 (0–4)	1 (0–5)	0.599
Nadir Hemoglobin g/dl, median (range)	8.7 (5.6-12.5)	12 (5.9-16.5)	**<0.0001**
Glucose mg/dl, median (range)	117.5 (70–354)	130 (80–497)	0.540
FFP administered, %	20	11.4	0.244
Platelets administered, %	26.7	15.2	0.167
24 hour hematoma volume expansion mL, median (range)	13 (1–192)	12.3 (0–402)	0.486
Hematoma evacuation, %	13.3	10.1	0.633
Intraventricular catheter placement	13.3	30.4	0.069
ICH location, %			0.347
IVH	0	1.3	
Basal ganglia	53.3	41.8	
Caudate	3.4	0	
Cerebellar	6.7	2.5	
Lobar	20	22.8	
Pons	3.3	10.1	
Thalamus	13.3	21.5	

*TIA* transient ischemic attack, *SBP* systolic blood pressure, *DBP* diastolic blood pressure, *GCS* Glasgow coma scale, *ICH* intracerebral hemorrhage, *FFP* fresh frozen plasma, *tPA* tissue plasminogen activator, *IVH* intraventricular hemorrhage.

ICH outcomes based on admission hemoglobin are shown in Table 2. Patients with low baseline hemoglobin had longer hospital lengths of stay than patients with normal hemoglobin (13.5 days vs. 7 days; p = 0.037). There were no significant differences in median discharge NIHSS (13 vs. 9; p = 0.527) or discharge mRS (4 vs. 4; p = 0.362) between the two groups.

**Table 2:** **Admission hemoglobin and outcomes from intracerebral hemorrhage**

	Low hemoglobin	Normal hemoglobin	p value
Length of Stay, median (range)	13.5 (1–86)	7 (1–40)	**0.037**
Discharge NIHSS, median (range)	13 (0–42)	9 (0–42)	0.527
Discharge mRS, median (range)	4 (2–6)	4 (1–6)	0.362

NIHSS: National Institute of Health Stroke Scale, mRS: modified Rankin Scale.

### Nadir hemoglobin

**Figure 1:**
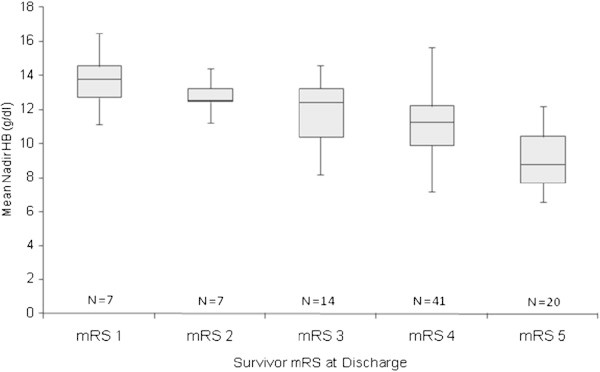
**Nadir hemoglobin values and functional outcome at discharge in surviving ICH patients.**

The relationship between nadir hemoglobin and functional outcome from ICH was examined using cumulative logit models. Lower nadir hemoglobin was associated with poor functional outcome in the unadjusted cumulative logit model (OR = 1.58, 95% CI 1.31-1.90, p < 0.0001). Lower nadir hemoglobin remained a significant predictor of poor outcome after adjusting for age, baseline NIHSS, transfusion, and length of stay (OR = 1.43, 95% CI 1.06-1.94, p = 0.0213) (Table 3). Baseline NIHSS was also a significant predictor of poor outcome (OR = 0.791, 95% CI 0.729-0.858, p < 0.0001) following ICH in the adjusted model. Increasing discharge mRS scores were associated with lower nadir hemoglobin values (Figure 1). Lower nadir hemoglobin was not a predictor of in-hospital mortality on logistic regression analysis (OR 1.12, 95% CI 0.917-1.364, p = 0.271) in a crude model.

**Table 3:** **Adjusted cumulative logit model using nadir hemoglobin to predict poor outcome (modified Rankin Scale 5–6)**

	Odds ratio	95% CI	p value
Nadir Hemoglobin	1.429	1.055-1.938	**0.0213**
Age	0.969	0.932-1.007	0.1087
Baseline NIHSS	0.791	0.729-0.858	**<0.0001**
Transfusion	0.194	0.033-1.132	0.0685
Length of Stay	0.985	0.944-1.028	0.4982

*NIHSS* National Institute of Health Stroke Scale.

### Transfusion

Outcome analysis was repeated to examine the impact of pRBC transfusion. The unadjusted cumulative logit model showed that patients who received a transfusion had 9 times greater odds of having a discharge mRS 5–6 (OR 9.37, 95% CI 2.84-30.88, p = 0.0002) compared with patients who did not receive transfusion. When age, nadir hemoglobin, admission ICH score, and intubation at any time were adjusted for, this was no longer statistically significant but still showed a positive association (OR 4.01, 95% CI 0.64-25.32, p = 0.1392). Transfusion was associated with in-hospital mortality in the crude logistic regression analysis (OR 2.99, 95% CI 1.01-8.85, p = 0.0473), but did not remain an independent predictor after multivariable analysis (OR 6.88, 95% CI 0.68-69.70, p = 0.1208).

## Discussion

We found that patients with poor functional outcome at discharge (mRS 5–6) had lower nadir hemoglobin values than patients with good functional outcome. Patients with low admission hemoglobin values had longer lengths of stay, but only nadir hemoglobin remained an independent predictor of LOS after controlling for other factors impacting outcome. After controlling for age, NIHSS, and admission hemoglobin, transfusion did not remain a significant predictor of NIHSS at discharge and poor functional outcome at discharge.

Diedler et al. found that patients with poor outcome at discharge (mRS 4–6) had lower admission and nadir hemoglobin values. (Diedler et al. 2010) In this study, mean hemoglobin was found to be an independent predictor of functional outcome at discharge in multivariable analysis but the impact of admission and/or nadir hemoglobin was not reported. Transfusion was not found to be a significant predictor of functional outcome (Diedler et al. 2010), which is supported by the results of the present study.

We did not find transfusion to negatively impact outcome; poor outcome may be a function of medical illness, and blood transfusion may serve as a marker for this. It remains unclear whether treatment of anemia with transfusion can improve outcome from ICH. Sheth et al. found transfusion was independently associated with decreased 30-day mortality after controlling for anemia and other known predictors of mortality in ICH. (Sheth et al. 2011) Further study is needed to assess this relationship. A randomized, prospective trial evaluating the threshold for transfusion treatment of anemia in ICH is warranted to clarify this relationship.

Our study is limited by a relatively small sample size and retrospective design. Treatment of anemia was not standardized, and there was variability in transfusion practices based on provider judgment. The group with normal hemoglobin had higher DBP and higher SBP despite this relationship not being statistically significant, which could have biased results in favor of the low hemoglobin group. This may have also contributed to the lack of association between admission hemoglobin and outcome. Further clarification could be obtained by following patients longitudinally to assess long-term functional outcomes. To our current knowledge, there are no prospective, randomized studies to evaluate anemia and transfusion in ICH.

## Conclusion

This study suggests that nadir hemoglobin, not admission hemoglobin, can be used to predict poor functional outcome in patients with ICH. Nadir hemoglobin is lowest in patients with baseline anemia and may serve as a surrogate marker for medically complex patients. Further research is warranted to evaluate the effects of anemia and treatment with pRBC transfusion on outcomes from acute ICH.

## Disclosures

The project described was supported by Award Numbers 5 T32 HS013852-10 from The Agency for Healthcare Research and Quality (AHRQ), 3 P60 MD000502-08S1 from The National Institute on Minority Health and Health Disparities (NIMHD), National Institutes of Health (NIH), and 13PRE13830003 from the American Heart Association (AHA). The content is solely the responsibility of the authors and does not necessarily represent the official views of the AHRQ, NIH, or AHA.
